# Tensile‐Strained RuO_2_ Loaded on Antimony‐Tin Oxide by Fast Quenching for Proton‐Exchange Membrane Water Electrolyzer

**DOI:** 10.1002/advs.202201654

**Published:** 2022-06-19

**Authors:** Bing Huang, Hengyue Xu, Nannan Jiang, Minghao Wang, Jianren Huang, Lunhui Guan

**Affiliations:** ^1^ CAS Key Laboratory of Design and Assembly of Functional Nanostructures Fujian Key Laboratory of Nanomaterials Fujian Institute of Research on the Structure of Matter Chinese Academy of Sciences Fuzhou 350000 China; ^2^ Collage of Materials Science and Opto‐Electronic Technology University of Chinese Academy of Sciences Beijing 100049 China; ^3^ Institute of Biopharmaceutical and Health Engineering Tsinghua Shenzhen International Graduate School Tsinghua University Shenzhen 518055 China

**Keywords:** electrochemical catalysis, oxygen evolution reaction, proton‐exchange membrane water electrolyzer, RuO_2_, water electrolysis

## Abstract

Future energy demands for green hydrogen have fueled intensive research on proton‐exchange membrane water electrolyzers (PEMWE). However, the sluggish oxygen evolution reaction (OER) and highly corrosive environment on the anode side narrow the catalysts to be expensive Ir‐based materials. It is very challenging to develop cheap and effective OER catalysts. Herein, Co‐hexamethylenetetramine metal–organic framework (Co‐HMT) as the precursor and a fast‐quenching method is employed to synthesize RuO_2_ nanorods loaded on antimony‐tin oxide (ATO). Physical characterizations and theoretical calculations indicate that the ATO can increase the electrochemical surface areas of the catalysts, while the tensile strains incorporated by quenching can alter the electronic state of RuO_2_. The optimized catalyst exhibits a small overpotential of 198 mV at 10 mA cm^−2^ for OER, and keeps almost unchanged after 150 h chronopotentiometry. When applied in a real PEMWE assembly, only 1.51 V is needed for the catalyst to reach a current density of 1 A cm^−2^.

## Graphical abstract

The tensile strained RuO_2_ nanorod catalyst loaded on antimony‐tin oxide (ATO) substrate is prepared by a fast‐quenching method. With multiple characterizations, it is proved that the tensile strain and ATO substrate are beneficial for the oxygen evolution reaction (OER). This work has demonstrated the OER enhancements in RuO_2_ when tensile strains are incorporated.

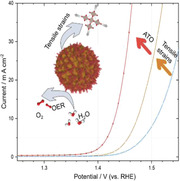

## 1 Introduction

Hydrogen energy can offer a promising way to reduce fossil energy usage and alleviate carbon dioxide emissions.^[^ ^1^ ^]^ Water electrolysis, consisting of two half‐reactions of the hydrogen evolution reaction (HER) and oxygen evolution reaction (OER), is regarded as one of the best solutions to hydrogen production.^[^ ^2^ ^]^ The HER is known to be more favorable in kinetics and mass transport,^[^ ^3^ ^]^ so it is more compulsory to develop OER catalysts for coupling industrial applications. In alkaline electrolytes, layered double hydroxides are active and stable enough to drive the sluggish OER.^[^ ^4^ ^]^ However, most of the reported catalysts suitable for alkaline OER are thermodynamically unstable when operated in acid electrolytes. For now, it is commonly accepted that proton‐exchange membrane water electrolyzers (PEMWE) can deliver much higher current densities than alkaline electrolyzers due to the intrinsic high ionic conductivity of the proton membrane.^[^ ^5^ ^]^ Therefore, PEMWE devices are more practicable for industrial usage. Nevertheless, the acidic media and high operating voltages in PEMWE have limited the anodic catalysts to be expensive Ir‐based materials.^[^ ^6^ ^]^ Beyond Ir‐based materials, much cheaper Ru‐based materials can also be employed for OER in acid media.^[^ ^7^ ^]^ Furthermore, Nørskov's volcano curve has indicated that the activity of RuO_2_ toward OER is located near the peak position.^[^ ^8^ ^]^ Though with relatively higher OER activity than IrO_2_, RuO_2_ cannot operate for a long time due to the formation of volatile RuO_4_, which tends to dissolve in the electrolyte.^[^ ^9^ ^]^

In previous reports, doping of foreign atoms is the mainstream for boosting the OER activity of RuO_2_.^[^ ^10^ ^]^ Indeed, Sargent et al. had done high‐throughput density functional theory (DFT) screenings and experimental trials to indicate that the Ru‐Ir‐Sr ternary oxide can achieve exceptionally high OER activity and stability.^[^ ^7b^ ^]^ However, the selective leaching of unstable elements (dopants) is essential for obtaining high OER activity.^[^ ^11^ ^]^ In real PEMWE devices, transition metal ions leached from the anode catalysts would irreversibly poison the proton membranes, thus degrading the electrolyzer performances.^[^ ^12^ ^]^ So alternative methods must be proposed to modify RuO_2_ without highlighting the doping of unstable atoms. To this end, strains can be introduced as positive enhancements for electrolysis.^[^ ^13^ ^]^ For instance, the self‐generated strained Pt skins outside the Pt‐M (transition metals) alloy can offer a much higher mass activity than the pristine Pt in ORR.^[^ ^14^ ^]^ While for the OER, Yang et al. prepared a strained iridium oxide catalyst based on lattice mismatches, and the strained sample exhibited a mass activity of 3.7 times higher than that of bare iridium oxide.^[^ ^15^ ^]^ Strains would cause electron structure modifications.^[^ ^16^ ^]^ Since the electron structure is closely involved in the binding with oxygen intermediates (*OH, *O, and *OOH), changes of it would tune the catalytic activity.^[^ ^16^, ^17^ ^]^ However, studies related to strain‐influenced RuO_2_ activity toward OER are still missing due to the complex preparation procedures like epitaxial growths of thin films.^[^ ^18^ ^]^ So easy methods to induce strains in RuO_2_ and systematic investigations on it are urgently needed.

Here, we report tensile strained RuO_2_ nanorods growing on antimony‐tin oxide (ATO) particles using the Co‐hexamethylenetetramine metal‐organic framework (Co‐HMT) and fast‐quenching treatment. The tensile strains were introduced on RuO_2_ nanorods due to the fast‐quenching process instead of lattice mismatches between ATO and RuO_2_. As a result, the modified electronic states promote the OER activity with an overpotential as low as 198 mV at 10 mA cm^−2^. Moreover, the catalyst shows no apparent degradations after 12 h chronopotentiometry on the carbon electrode or 150 h chronopotentiometry on the Ti felt electrode. When applied in PEMWE devices, only 1.51 V (IR‐free) is needed to drive a current density of 1 A cm^−2^. Our work has demonstrated that tensile strains can be employed as a positive enhancement for OER of RuO_2_, thus would inspire future OER catalyst designs.

## 2 Results and Discussion

**Figure 1:**
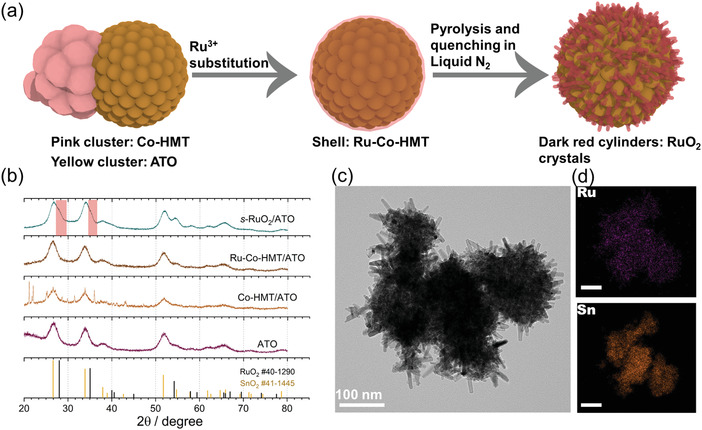
a) The schematic illustration of the synthesis procedures. b) The XRD patterns of ATO, Co‐HMT/ATO, Ru‐Co‐HMT/ATO, and *s*‐RuO_2_/ATO. The red areas inserted indicate the bumps induced by the existence of RuO_2_. c) The TEM image of *s*‐RuO_2_/ATO. d) The elements mapping images of *s*‐RuO_2_/ATO. The scale bars inserted are 100 nm.

As illustrated in **Figure** **1**a, the tensile‐strained RuO_2_ loaded on ATO was prepared via ion substitutions, pyrolysis, and fast quenching. The ATO was prepared by the method presented in the previous report.^[^ ^19^ ^]^ The Co‐HMT was first formed in ATO dispersions by adding the Co salt and hexamethylenetetramine, and the self‐assembled product was noted as Co‐HMT/ATO. From the scanning electron microscope (SEM) image shown in Figure S1 (Supporting Information), it is found that the Co‐HMT exists as spindle‐like crystals together with irregular ATO nanoparticles. The as‐prepared Co‐HMT/ATO composites were then subscribed to Ru^3+^ substitutions, where the RuCl_3_•3H_2_O/Co‐HMT mass ratios are fixed to be around 1:1 to ensure sufficient ion replacements. After Ru^3+^ substitutions, no separated Co‐HMT can be observed (Figure S2, Supporting Information), indicating the destruction of Co‐HMT structures during the substitution process (noted as “Ru‐Co‐HMT/ATO”). The XRD patterns in Figure 1b also prove that no pure Co‐HMT residuals are retained in Ru‐Co‐HMT/ATO. Besides, the transmission electron microscopy (TEM) and corresponding elementals mapping images (Figure S3, Supporting Information) show that the Ru elements tend to reside on the ATO surfaces, implying that the Co‐HMT might undergo dissolution and redeposition in the Ru^3+^ substitution process. In the last step, final pyrolysis was conducted to transform the Ru species into RuO_2_. In order to incorporate tensile strains into the RuO_2_, a fast‐quenching process was performed.^[^ ^20^ ^]^ In detail, the powder product was immediately taken out from a hot furnace and quenched in liquid N_2_ to preserve the residual stresses. After quenching, the final sample was collected and named *s*‐RuO_2_/ATO (“*s*” refers to “strained”).

In the X‐ray diffraction (XRD) patterns of *s*‐RuO_2_/ATO, no prominent diffraction peaks attributed to RuO_2_ are presented. Conversely, only small bumps near the ATO peaks (SnO_2_) can be identified (Figure 1b). As shown in the TEM images (Figure 1c), *s*‐RuO_2_/ATO exhibits a nanorod morphology, totally different from the initial ATO (Figures S4 and S5, Supporting Information). Similar morphology has been reported in Co‐doped RuO_2_.^[^ ^10a^ ^]^ Furthermore, elementals mapping images (Figure 1d) show that Ru elements are more prone to distribute on the outside while Sn elements are on the inside, proving that the nanorods outside are RuO_2_ crystals instead of ATO. The nanorod structures of *s*‐RuO_2_/ATO can favor the maximum exposure of OER active sites.

Apart from *s‐*RuO_2_/ATO, another three samples including *n*‐RuO_2_/ATO (without quenching process, “*n*” refers to “normal”), *s*‐RuO_2_ (without ATO substrate), and *n*‐RuO_2_ (without quenching process and ATO substrate) were prepared as counterpart references. As shown in Figure S6 (Supporting Information), n‐RuO_2_/ATO shows identical morphology to *s‐*RuO_2_/ATO, implying that the quenching process does not change the overall morphology. Similar morphologies can also be observed between *s*‐RuO_2_ and *n*‐RuO_2_ (Figure S7, Supporting Information)_._ However, samples without ATO substrates show thicker and broader nanorods. Some of the nanorods are even broader than 30 nm, much bigger than samples with ATO substrates (Figure S8, Supporting Information), indicating that ATO substrates can reduce the sizes of RuO_2_ crystals. It is easy to conjecture that thin‐layer precursors containing Ru elements covered on ATO surfaces have fewer sources to contact and grow, so relatively smaller RuO_2_ nanorods can grow on the ATO substrates. The Ru and Co contents in *s‐*RuO_2_/ATO checked by inductively coupled plasma mass spectrometry (ICP‐MS) are 19.5 and 2.4 wt%, and similar results are also obtained from *n‐*RuO_2_/ATO provided by the same precursors (Table S1, Supporting Information). Here, it should be noted that small fractions of Co residuals are inevitable due to Co‐HMT precursors. Since several reports have clarified that Co dopants positively influence the OER,^[^ ^10^, ^21^ ^]^ we have tentatively kept the Ru/Co ratios during the substitution processes for a rational comparison.

**Figure 2:**
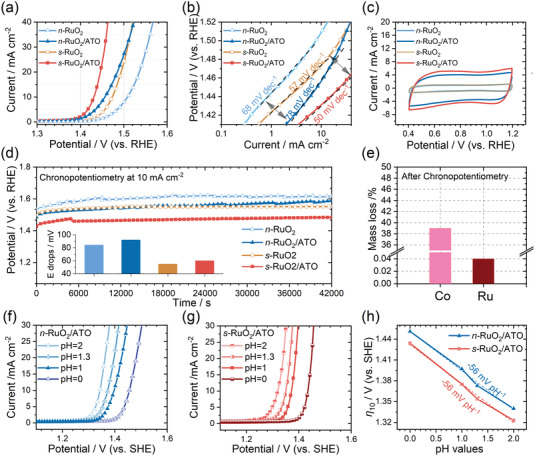
a) The LSV curves, b) The Tafel slopes, c) The CV curves, and d) The chronopotentiometry curves of *n*‐RuO_2_, *n*‐RuO_2_/ATO, *s*‐RuO_2_, and *s*‐RuO_2_/ATO. e) The mass loss percentages evaluated from ICP‐MS results in the electrolyte after 12 h chronopotentiometry of *s*‐RuO_2_/ATO. f) The LSV curves of *n*‐RuO_2_/ATO recorded at pH = 2, 1.3, 1, and 0. g) The LSV curves of *s*‐RuO_2_/ATO recorded at pH = 2, 1.3, 1, and 0. h) The *η*_10_ from f,g) recorded at different pH values for *n*‐RuO_2_/ATO and *s*‐RuO_2_/ATO.

The electrochemical OER performances were first assessed on a three‐electrode rotating ring electrode configuration in 0.1 m HClO_4_ solution. For comparison, the load masses of Ru for all samples keep at 0.19 mg cm^−2^. In **Figure** **2**a; and Figure S9 (Supporting Information), the linear sweep voltammetry (LSV) curves are plotted in the range of 1.3–1.6 V (vs reversible hydrogen electrode (RHE)). As can be seen, the optimized *s‐*RuO_2_/ATO exhibits an overpotential of 198 mV at the current density of 10 mA cm^−2^ (*η*_10_), which is much lower than that of *n*‐RuO_2_/ATO (*η*_10_ = 224 mV), indicating that the quenching process is beneficial for boosting the OER activity. Here, the reported *η*_10_ of *s‐*RuO_2_/ATO is lower than most previous works (Table S2, Supporting Information). Additionally, the sample without the ATO substrate (*s‐*RuO_2_) exhibits lower activity (*η*_10_ = 241 mV) than *n*‐RuO_2_/ATO, demonstrating that the ATO substrates are also vital for enhancing the OER activity. Identical to the above assumption, the *n*‐RuO_2_ without quenching process and ATO substrate show the worst OER activity (*η*_10_ = 276 mV).

The LSV curves were further plotted on the logarithm axis. As shown in Figure 2b, the Tafel slopes for *s‐*RuO_2_/ATO, *n‐*RuO_2_/ATO, *s‐*RuO_2_, *n‐*RuO_2_ are 50, 78, 57, and 68 mV dec^−1^, respectively. The trend summarized from the obtained Tafel slopes is that the quenching process could reduce the Tafel slope. Besides, the quenched samples seem to exhibit similar Tafel slopes, and so do the unquenched samples. Since the Tafel slopes reflect the intrinsic microkinetic in electrochemical processes, we suggest that the quenching process could alter the catalyst's binding strengths with the OER intermediates, thus rendering different coverages of oxygen intermediates.^[^ ^22^ ^]^

In order to investigate the intrinsic activity of the samples, we estimated the electrochemical surface areas (ECSA) by calculating the double‐layer capacitances based on the cyclic voltammetry (CV) measurements between 0.4 and 1.2 V (vs RHE) under continuous N_2_ bubbling. In Figure 2c; and Figure S10 (Supporting Information), it is observed that the ECSAs of *s‐*RuO_2_/ATO and *n‐*RuO_2_/ATO are nearly four times larger than those of *s‐*RuO_2_ and *n‐*RuO_2_. Though the total load masses for samples with the ATO substrates are much higher, the recorded CV curve of bare ATO at the load mass equal to the quantity in *s‐*RuO_2_/ATO indicates that the ATO is not the main factor for the enhanced ECSA (Figure S11, Supporting Information). As discussed above, the ATO can decrease the crystal sizes of RuO_2_, which might be the primary reason for the enhanced ECSA after using ATO as substrates. Besides, we also observe that the quenching process also improves ECSAs from the differences between quenched and unquenched samples. To clarify if the enhanced OER activity originated from the enhanced ECSAs, we normalized the LSV curves by ECSA capacitances in Figure S12 (Supporting Information). As can be seen, these differences between samples with and without ATO substrates are diminished. Nevertheless, for the quenched and unquenched counterparts, a significant difference still exists.

As mentioned above, the Co dopants are inevitable. Since the Ru/Co ratios are similar in samples, it can be deduced that the above conclusions about the positive effects of ATO and quenching are reasonable. To extend the quenching effect, we have also prepared RuO_2_ from the fast‐quenching process with no Co involvement (see detailed information in the Experimental Section). As shown in Figure S13 (Supporting Information), the quenching step enhances the OER performances of RuO_2_, proving its transferability to undoped RuO_2_.

The long term‐stability tests were performed at 10 mA cm^−2^ for 12 h and presented in Figure 2d. As can be seen, after 12 h chronopotentiometry, these samples show increased overpotentials at different degrees. The increased overpotentials were plotted in the inserted image of Figure 2d. Intriguingly, these samples with ATO as substrates show no significant differences compared to their counterparts, evidencing that the ATO has limited effects on improving the stability of RuO_2_. However, previous studies have presented that the ATO substrate can improve the stability of IrO_2_.^[^ ^19^, ^23^ ^]^ Given the similarities in rutile structures between RuO_2_ and IrO_2_, we believe that the insufficient contacts between RuO_2_ and ATO may result in the limited effects of ATO in our experiment. Nevertheless, samples with quenching processes exhibit improved stability compared to their counterparts, proving that quenching can be used as a positive factor to enhance the stability of RuO_2_. Combining with the Tafel slopes and ECSAs analyzed above, we tentatively suggest that the ATO substrate can increase the exposed active sites, and the quenching process can alter the intrinsic characteristics of Ru sites. In addition, the CV curve of *s‐*RuO_2_/ATO after chronopotentiometry shows a 13% increment in ECSA. (Figure S14, Supporting Information). As evidenced by the ICP‐MS results (Figure 2e), we attribute the increased ECSA to the leaching of Co atoms.

The glass carbon electrode is not suitable for more extended chronopotentiometry tests due to the detachment of active materials and oxidation of the carbon electrode. Further evaluations of *s‐*RuO_2_/ATO's stability were performed on Ti felt electrode. There were no apparent attenuations of the potential after a continuous 150 h chronopotentiometry test (Figure S15, Supporting Information).

The LSV curves recorded at different pH values are presented in Figure 2f,g on the standard hydrogen electrode (SHE) scales. As can be seen, the LSV curves gradually move to higher potentials under lower pH values. In Figure 2h, we observed a clear pH‐dependent *η*_10_ with the same slopes of −56 mV pH^−1^ (concerted proton‐electron transfer: −59 mV pH^−1^), demonstrating the absence of the lattice oxygen mechanism (LOM) pathway. The LOM pathway is not beneficial for the structural stability of the catalysts due to the collapse of the metal–oxygen framework. The absence of the LOM pathway can partly explain the excellent stability of *s*‐RuO_2_/ATO.^[^ ^24^ ^]^ The above results demonstrate the synergic positive effects of the ATO substrate and quenching process toward OER, but pertinent proofs are still needed to unravel the mechanism.

**Figure 3:**
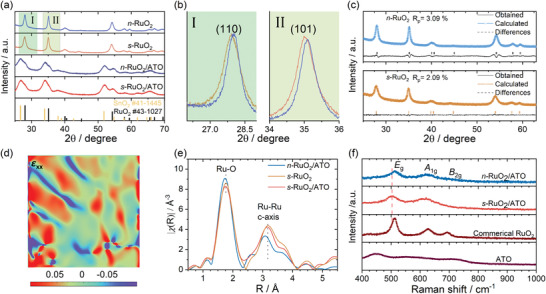
a) The XRD patterns of *n*‐RuO_2_, *n*‐RuO_2_/ATO, *s*‐RuO_2_, and *s*‐RuO_2_/ATO. b) The magnified regions XRD patterns of *n*‐RuO_2_ and *s*‐RuO_2_. c) Rietveld refinements of the XRD patterns for *n*‐RuO_2_ and *s*‐RuO_2_. d) The GPA image of *s‐*RuO_2_/ATO for axial strain (*ɛ*_xx_). e) The Ru K‐edge FT‐EXAFS spectra of *n*‐RuO_2_/ATO, *s*‐RuO_2_, and *s*‐RuO_2_/ATO after phase corrections. f) The Raman spectra of *n*‐RuO_2_/ATO, *s*‐RuO_2_/ATO, Commercial RuO_2_, and ATO.

The XRD is sensitive to subtle differences in crystal structures. To carefully screen the differences between samples, prolonged scans with 0.5° min^−1^ were used. As shown in **Figure** **3**a, *s‐*RuO_2_/ATO and *n‐*RuO_2_/ATO show two sets of diffraction peaks, which can be assigned to SnO_2_ (PDF No: 41‐1445) and RuO_2_ (PDF No: 40‐1290). The peaks of RuO_2_ in *s‐*RuO_2_/ATO and *n‐*RuO_2_/ATO are heavily overlapped with peaks from SnO_2_, and only small bumps at higher degrees can be distinguished. Additionally, the diffraction peaks of *s*‐RuO_2_ and *n*‐RuO_2_ concord very well with the RuO_2_. It can be seen that the diffraction peaks of RuO_2_ in *s*‐RuO_2_ and *n*‐RuO_2_ are much more pronounced and narrower than *s‐*RuO_2_/ATO and *n‐*RuO_2_/ATO (Figures S16 and S17, Supporting Information), confirming that the crystal sizes in samples without the ATO substrates are larger, and agreeing well with the above discussions. When closely inspecting the peak positions (Figure 3b), the corresponding peaks are negatively shifted for *s*‐RuO_2_ compared to *n*‐RuO_2_. Based on the diffraction equation, lower diffraction angles mean expanded lattice parameters, so it can be deduced that tensile strains are incorporated in *s*‐RuO_2_.^[^ ^25^ ^]^ The XRD patterns after Rietveld refinements indicate that the lattice parameters of *s*‐RuO_2_ are slightly larger than *n*‐RuO_2_ (Figure 3c; and Table S3, Supporting Information). The samples with ATO substrates are not suitable for Rietveld refinements due to the very weak diffraction peaks of the RuO_2_ phase. However, the boarding of lattice parameters in *s*‐RuO_2_ can hint that the induced tensile strains are related to the quenching process. Besides, when *s*‐RuO_2_ was further annealed at 350 °C and cooled down to room temperature naturally, these characteristic peaks of RuO_2_ moved back to positions of *n*‐RuO_2_ due to the release of residual strains (Figure S18, Supporting Information).

High‐resolution TEM images can provide direct evidence for the differences existing in crystal structures. As shown in Figures S19 and S20 (Supporting Information), the interplanar spacings of (110) were obtained by averaging 8 accumulations. Similar to Rietveld refinements (Table S3, Supporting Information), the interplanar spacings of (110) of rutile‐structured RuO_2_ are 3.20, 3.23, and 3.23 Å for *n‐*RuO_2_/ATO, *s‐*RuO_2_/ATO, and *s‐*RuO_2_, respectively. Nevertheless, a large number of grain boundaries and stacking faults are also frequently observed in *s‐*RuO_2_/ATO and *s‐*RuO_2_ (Figure S21, Supporting Information), which could be related to the fast quenching process.^[^ ^26^ ^]^ Geometric phase analysis (GPA) was also performed near these defective regions.^[^ ^27^ ^]^ As shown in Figure 3d; and Figures S22 and S23 (Supporting Information), s‐RuO_2_/ATO and *s‐*RuO_2_ process high distortions in these regions. In terms of effects from ATO, no evidence of its influences on crystal structures can be identified. As *s‐*RuO_2_/ATO exhibits excellent long‐term stability, post‐OER (On glass carbon electrode) TEM characterization is also critical to assess the structural integrity. Figure S24 (Supporting Information) shows that tensile strains are preserved after 12 h chronopotentiometry, demonstrating the excellent stability of *s‐*RuO_2_/ATO.

X‐ray absorption spectroscopy (XAS) and Raman spectrum were also employed to investigate the incorporated tensile strains presented in *s‐*RuO_2_/ATO. In the Fourier transformed extended X‐ray absorption fine structure (FT‐EXAFS), the first and second shells of *s*‐RuO_2_/ATO and *s*‐RuO_2_ have longer radial distances than *n*‐RuO_2_/ATO (Figure 3e). The longer radial distances observed in FT‐EXAFS indicate the existence of tensile strains.^[^ ^26^ ^]^ It should be mentioned that the scattering paths in FT‐EXAFS do not identically correspond to the real atomic distances due to multiple scattering.^[^ ^28^ ^]^ Hence, further discussions using lattice parameters are based on the results from TEM and XRD.

According to the previous reports, the *E*_g_ peak tends to redshift for the tensile‐strained RuO_2_ in the Raman spectrum.^[^ ^29^ ^]^ As expected, the *E*_g_ peaks of *s*‐RuO_2_/ATO and *s‐*RuO_2_ are shifted to a lower wavenumber than *n*‐RuO_2_/ATO (Figure 3f; and Figure S25, Supporting Information). In conclusion, the diffractometry and spectroscopy methods can testify that the quenching process could induce tensile strains in the RuO_2_. However, we do not observe enough evidence that ATO could influence the crystal structures of RuO_2_ in the above characterizations, possibly due to the insufficient contacts between RuO_2_ nanorods and ATO substrates.

**Figure 4:**
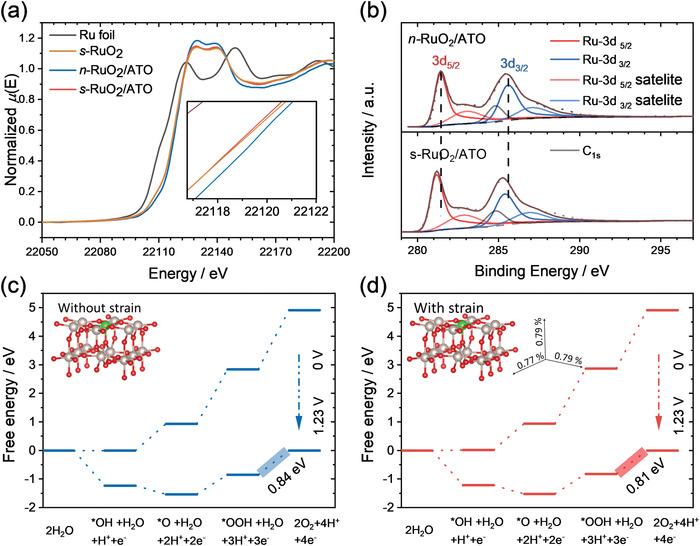
a) The XANES spectra of Ru foil, *n*‐RuO_2_/ATO, *s*‐RuO_2_, and *s*‐RuO_2_/ATO. b) The XPS‐Ru_3d_ spectra of *n*‐RuO_2_/ATO and *s*‐RuO_2_/ATO. The peaks used for Ru 3d fitting are asymmetric, while the carbon C1s peaks are symmetric.^[^ ^30^ ^]^ The same parameters were used to fit all the Ru 3d peaks. c,d) The free energy diagrams of RuO_2_ and strained RuO_2_ calculated on the coordinatively unsaturated sites (CUS, colored with green). The inserted axis gives the strain values in different directions of the strained RuO_2_.

The chemical environments were characterized by the X‐ray absorption near‐edge structure (XANES) and X‐ray photoelectron spectroscopy (XPS). In the XANES spectra (**Figure** **4**a), the samples with tensile strains (*s*‐RuO_2_ and *s*‐RuO_2_/ATO) show slightly lower absorption edges than *n*‐RuO_2_/ATO, indicating the lower valence states induced by tensile strains. Similar negative shifts are also observed in the XPS Ru 3d regions (Figure 4b; and Figure S26, Supporting Information), supporting the results from XANES. The Ru site in high chemical valence states tends to be leached out, compromising the stability of crystal structures.^[^ ^9^ ^]^ So, we tentatively attribute that the stability of *s‐*RuO_2_/ATO benefits from the lower valence states of Ru sites. Besides, there is no sufficient evidence of the interactions between ATO and RuO_2_ that can be identified due to the minor difference in *s*‐RuO_2_ and *s*‐RuO_2_/ATO (XANES and XPS results), suggesting that tensile strains are the leading causes of the modified electronic states.

DFT was employed to investigate the  effect of tensile strains. Two relatively simple models with no consideration of cobalt dopants and involvements of linear relation breaking sites (Insets in Figure 4c,d). Four elemental steps of OER are considered in the calculation

(1) $$M+2H_{2}O=\text{M-OH}+H_{2}O+H^{+}+e^{-}$$

(2) $$\text{M-OH}+H_{2}O+H^{+}+e^{-}=\text{M-O}+H_{2}O+2H^{+}+2e^{-}$$

(3) $$\text{M-O}+H_{2}O+2H^{+}+2e^{-}=\text{M-OOH}+3H^{+}+3e^{-}$$

(4) $$\text{M-OOH}+3H^{+}+3e^{-}=M+O_{2}+4H^{+}+4e^{-}$$

As shown in Figure 4c,d, the barrier of the limiting step of OER decreases after the introduction of tensile strains into RuO_2_. Besides, the absorption energies of oxygen intermediates are weakening on the tensile‐strained RuO_2_. According to Nørskov's results, relatively lower binding strengths of oxygen intermediates could improve the OER in RuO_2_.^[^ ^8a^ ^]^ When considering the bridge‐O coverages on the surfaces of RuO_2_ in DFT calculations, a similar trend with decreased limiting step after applying the tensile strains is also observed (Figures S27 and S28, Supporting Information). Notably, the limiting step on the strained RuO_2_ with bridge‐O coverage is smaller and much closer to the experimental results.

**Figure 5:**
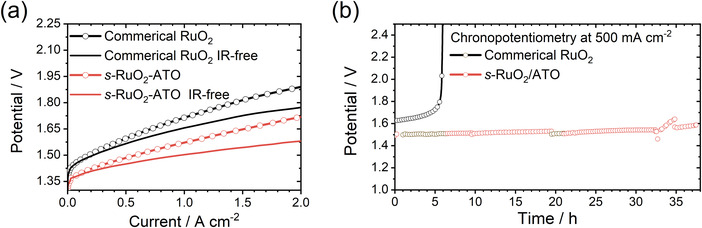
a) The LSV curves of *s‐*RuO_2_/ATO and commercial RuO_2_ in PEMWEs. b) The chronopotentiometry curves (without IR corrections) at 500 mA cm^−2^ of *s‐*RuO_2_/ATO and commercial RuO_2_ in PEMWEs.

As a model catalyst, *s‐*RuO_2_/ATO, incorporated with the ATO substrate and tensile strains, was used as the anode material in PEMWE. Detailed configurations of the PEMWE assembly can be found in Figure S29 (Supporting Information). The PEMWEs were tested at 80 °C with a water flow of 100 mL min^−1^ to the anode only. **Figure** **5**a shows the recorded LSV curves. As shown, the *s*‐RuO_2_/ATO has displayed a remarkably high activity with the potential of only 1.51 V at 1 A cm^−2^ (IR‐free), far surpassing the commercial RuO_2_. Furthermore, the assembled PEMWEs were operated at 0.5 A cm^−2^. As shown in Figure 5b, the PEMWE using commercial RuO_2_ as the anode cannot work anymore after 5 h. The PEMWE using *s‐*RuO_2_/ATO as the anode shows no significant decline even after 40 h continuous operation. As discussed above, the high efficiency and robustness of the PEMWE using *s‐*RuO_2_/ATO as the anode can be ascribed to the incorporations of ATO substrate and tensile strains.

## 3 Conclusions

In summary, we have successfully prepared a catalyst with strained RuO_2_ nanorods loaded on the ATO substrate by employing the ATO as substrate and the quenching process to induce tensile strains. The ATO substrate enhances the ECSA of RuO_2_, while tensile strains induced by quenching can modify the electronic states of RuO_2_. Synergetic effects from the ATO and tensile strains enable *s*‐RuO_2_/ATO to be one of the best OER catalysts with only 198 mV overpotential at 10 mA cm^−2^, and it is kept almost unchanged after 150 h chronopotentiometry. In addition, the PEMWE assembled by using *s*‐RuO_2_/ATO as anode also displays remarkably high efficiency and stability. This work demonstrated that tensile strains in RuO_2_ would positively influence the OER activity, and present a new perspective toward future catalyst designs.

## 4 Experimental Section

### Preparations of ATO

The ATO was synthesized following previous reports with slight modifications.^[^ ^31^ ^]^ First, 1.28 g dodecylamine (Adamas) was mixed with 65 mL ethanol and 160 mL deionized water, then stirred for 3 h to form an emulsion. At the same time, antimony triacetate (0.48 g, Macklin) and SnCl_4_ (2.15 mL, Macklin) were added into 20 mL ethanol. Then, 20 mL of the metal contained ethanol were slowly added into the above emulsion, followed by stirring for 1 h. After that, the above solution was then slowly added to 200 mL of 1.5 × 10^−3^ m NH_3_H_2_O solution and kept stirred for 1 h. The prepared solution was then transformed into an oil bath at 80 °C for 72 h. Then the precipitate was collected by centrifugation (5000 rpm, 10 min) and washed with water five times. The obtained wet product was dispersed in 60 mL water and transformed into a 100 mL autoclave at 120 °C for 24 h. The as‐prepared product was collected by centrifugation (5000 rpm, 10 min) and washed with water five times. Finally, the gray powder was calcinated in air at 400 °C for 3 h.

### Preparations of Co‐HMT/ATO

First, 200 mg of the prepared ATO was dispersed in 50 mL of isopropanol by sonication for 4 h. Then, 197 mg cobalt nitrate hexahydrate (Macklin) was dissolved into the ATO dispersion. The hexamethylenetetramine (HMT) (Sinopharm) solution was prepared by adding 950 mg HMT into a mixture of 2 mL water and 5 mL ethanol. Then, the HMT solution was dropwise added into ATO dispersion to initialize the formation of the Co‐HMT metal–organic framework. After reaction for 30 min, the sample was collected by centrifugation (5000 rpm, 5 min) and washed with isopropanol five times. Then, the sample was dried in a vacuum condition.

### Preparations of Ru‐Co‐HMT/ATO

The prepared 100 mg Co‐HMT/ATO was dispersed in 5 mL tetrahydrofuran by sonication for 10 min. Then 60 mg of the RuCl_3_• 3H_2_O (Adamas) was added, followed by a 24 h vigorous stir. The final sample was collected by centrifugation (10 000 rpm, 10 min) and washed with tetrahydrofuran two times. Then, the sample was dried in a vacuum condition.

### Preparations of *s*‐RuO_2_/ATO

The prepared Ru‐Co‐HMT/ATO was annealed in air at 350 °C for 2 h in a muffle furnace. After 2 h annealing, the sample was quickly taken out and immersed in liquid N_2_ (−196 °C) to finish the quenching process.

### Preparations of *n*‐RuO_2_/ATO

Sample of *n‐*RuO_2_/ATO were prepared similar to *s‐*RuO_2_/ATO except without the last quenching step. In detail, the sample was cooled down to room temperature inside the muffle furnace in nature.

### Preparations of *s*‐RuO_2_

The sample of *s‐*RuO_2_ was prepared similar to *s‐*RuO_2_/ATO except without the addition of ATO. To achieve a similar substitution degree of Co^2+^ by Ru^3+^, the mass ratio of Co‐HMT to RuCl_3_•3H_2_O is setted to be 1:1.

### Preparations of *n*‐RuO_2_

Sample of *s‐*RuO_2_ was prepared similar to *s‐*RuO_2_/ATO except without the addition of ATO and quenching step.

### Preparations of EG‐*s*‐RuO_2_ and EG‐*n*‐RuO_2_

The Ru nanoparticles were first synthesized by oil bathing the mixture of 60 mg RuCl_3_ and 30 mL ethylene glycol at 190 °C under stirring for 30 min. Then, the obtained Ru nanoparticles were collected by centrifugation and washed with ethanol and water several times. Later, the Ru nanoparticles were annealed (with a quenching step for *EG‐s‐*RuO_2_) in the same condition as *s‐*RuO_2_ and *n‐*RuO_2_.

### Preparations of *s*‐RuO_2_/C and *n*‐RuO_2_/C

30 mg Ketjen Black were mixed with 100 mg RuCl_3_• 3H_2_O in 15 mL H_2_O by simple sonication for 1 h. After that, the mixture was free‐dried. Then the same annealing process mentioned above was used to synthesize *s‐*RuO_2_/C and *n‐*RuO_2_/C.

### Physical Characterizations

X‐ray powder diffraction spectroscopy was recorded on Miniflex600 (Rigaku) with Cu K_*α*_ X‐ray (*λ* = 0.154 nm). Scanning electron microscope images were obtained in a Su‐8010 electron microscope. Transmission electron microscope (TEM) images were obtained in JEOL 2100F (JEOL). XAS spectra were collected at Shanghai Synchrotron Radiation Facility (SSRF) and analyzed by Athena software. The Raman spectra were recorded in the Horiba Jobin Yvon LabRAM ARAMIS system equipped with 633 nm light. Before recording the Raman spectra, the system was calibrated using a Si plate. XPS spectra were measured on ESCALAB 250Xi (ThermoFisher) and calibrated to adventitious C_1s_ peak at 284.8 eV.

### Theoretical Calculations

All spin‐polarized DFT calculations for periodic material systems were performed with the Vienna Ab initio simulation package (VASP)^[^ ^32^ ^]^ with the projector‐augmented wave (PAW) method. The exchange‐correlation function was handled using the generalized gradient approximation (GGA) formulated by the Revised Perdew–Burke–Ernzerhof (RPBE). The van der Waals (vdW) interactions are described with the DFT‐D3 method in Grimme's scheme. The interaction between the atomic core and electrons was described by the projector augmented wave method. The plane‐wave basis set energy cutoff was set to 500 eV. The Brillouin zone was sampled with a 3 × 3 × 1 grid centered at the gamma (Γ) point for geometry relaxation. The perfect RuO_2_ bulk was fully optimized using 5 × 5 × 5 k‐point Gamma (Γ) centered Monkhorst‐Pack mesh sampling. The RuO_2_ facets were modeled using a four atomic layer 1 × 2 supercell slab with 6.22 × 6.39 Å in *x* and *y* directions, respectively. The RuO_2_ facets with strain were modeled with 6.27 × 6.44 Å in *x* and *y* directions, respectively. A 15 Å vacuum region, ensuring negligible lateral interaction of adsorbates. The bottom two atomic layers were kept frozen at the lattice position. All structures with a dynamic magnetic moment were fully relaxed to optimize without any restriction until their total energies were converged to < 1×10^−6^ eV, and the average residual forces were < 0.02 eV Å^−1^. Moreover, the Gibbs free energy calculation is operated with the computational hydrogen electrode (CHE) model.^[^ ^33^ ^]^

### Electrochemical Tests

A three‐electrode configuration including a rotating disk electrode (RDE, working electrode), Ag/AgCl electrode (reference electrode), and graphite electrode (counter electrode) was used to record the linear sweep voltammetry (LSV), cyclic voltammetry (CV), and chronopotentiometry curves. The electrochemical workstation used was CHI 760E (CH Instruments). The ink was prepared by dispersing 5 mg sample into a mixture of 970 µL isopropanol and 30 µL Nafion (5 wt%), and sonicated for at least 1 h. The load masses on the RDE for all the samples are based on the Ru contents (ICP results), which were set to be 0.19 mg cm^−2^. The LSV and CV curves were recorded under N_2_ bubbling. Before the LSV and CV tests, at least 50 CV curves (0.4–1.2 V) were carried out to stabilize the catalyst. The LSV curves were recorded at 5 mV s^−1^, while the CV curves were at 100 mV s^−1^. Here, it should be mentioned that all the LSV curves were IR corrected automatically in CHI software.

Electrolytes of different pH were prepared from concentrated HClO_4_ (Sigma) by diluting. To reduce high ionic resistances in electrolytes with pH 1.3 and 2, certain K_2_SO_4_ (Sigma) was added to make the total ionic concentrations 0.1 m.

Proton‐exchange membrane water electrolyzer (PEMWE) was tested on Gamry (Gamry Instruments) Interface 5000E. The Membrane Electrode Assembly (MEA) was prepared using Nafion 212 by the Catalyst Coated Membrane (CCM) method with a geometric area of 2 cm^2^. The catalyst inks were prepared at the same I:C ratio (Nafion: Catalyst, mass ratio) used for three‐electrode measurements. In addition, the total load mass for the anode (*s‐*RuO_2_/ATO) was weighted to be 3.3 mg cm^−2^, while it was 0.6 mg cm^−2^ for the cathode (Pt/C, 20 wt%). It has to note that the PEMWE using commercial RuO_2_ also employed an anodic load mass of 3.3 mg cm^−2^. During the data recording, both the anode and cathode plate were heated to 80 °C. Besides, a water flow preheated to 80 °C at 100 mL min^−1^ was supplied to the anode side.

## Conflict of Interest

The authors declare no conflict of interest.

## Supporting information

Supporting Information

## Data Availability

Research Data are not shared.

## References

[1] X. Meng , A. Gu , X. Wu , L. Zhou , J. Zhou , B. Liu , Z. Mao , Int. J. Hydrog. Energy 2021, 46, 28887.

[2] a) A. Ursua , L. M. Gandia , P. Sanchis , Proc. IEEE 2012, 100, 410;

[3] S. Chatterjee , X. Peng , S. Intikhab , G. Zeng , N. N. Kariuki , D. J. Myers , N. Danilovic , J. Snyder , Adv. Energy Mater. 2021, 11, 2101438.

[4] M. A. Oliver‐Tolentino , J. Vázquez‐Samperio , A. Manzo‐Robledo , R. d. G. González‐Huerta , J. L. Flores‐Moreno , D. Ramírez‐Rosales , A. Guzmán‐Vargas , J. Phys. Chem. C 2014, 118, 22432.

[5] I. Vincent , D. Bessarabov , Renewable Sustainable Energy Rev. 2018, 81, 1690.

[6] a) K. Ayers , Curr. Opin. Chem. Eng. 2021, 33, 100719;

[7] a) C. Lin , J.‐L. Li , X. Li , S. Yang , W. Luo , Y. Zhang , S.‐H. Kim , D.‐H. Kim , S. S. Shinde , Y.‐F. Li , Z.‐P. Liu , Z. Jiang , J.‐H. Lee , Nat. Catal. 2021, 4, 1012;

[8] a) J. Rossmeisl , Z. W. Qu , H. Zhu , G. J. Kroes , J. K. Nørskov , J. Electroanal. Chem. 2007, 607, 83;

[9] a) K. Klyukin , A. Zagalskaya , V. Alexandrov , J. Phys. Chem. C 2019, 123, 22151; 10.1021/acs.jpcb.8b10980 30995047

[10] a) Y. Tian , S. Wang , E. Velasco , Y. Yang , L. Cao , L. Zhang , X. Li , Y. Lin , Q. Zhang , L. Chen , iScience 2020, 23, 100756; 31887659 10.1016/j.isci.2019.100756 PMC6941840

[11] a) Y. Chen , Y. Sun , M. Wang , J. Wang , H. Li , S. Xi , C. Wei , P. Xi , G. E. Sterbinsky , J. W. Freeland , A. C. Fisher , J. W. Ager , Z. Feng , Z. J. Xu , Sci. Adv. 2021, 7, eabk1788; 34890227 10.1126/sciadv.abk1788 PMC8664262

[12] a) S. Stiber , N. Sata , T. Morawietz , S. A. Ansar , T. Jahnke , J. K. Lee , A. Bazylak , A. Fallisch , A. S. Gago , K. A. Friedrich , Energy Environ. Sci. 2022, 15, 109;

[13] a) G. Meng , W. Sun , A. A. Mon , X. Wu , L. Xia , A. Han , Y. Wang , Z. Zhuang , J. Liu , D. Wang , Y. Li , Adv. Mater. 2019, 31, 1903616; 10.1002/adma.201903616 31373731

[14] C.‐L. Yang , L.‐N. Wang , P. Yin , J. Liu , M.‐X. Chen , Q.‐Q. Yan , Z.‐S. Wang , S.‐L. Xu , S.‐Q. Chu , C. Cui , H. Ju , J. Zhu , Y. Lin , J. Shui , H.‐W. Liang , Science 2021, 374, 459. 34672731 10.1126/science.abj9980

[15] W. Sun , Z. Zhou , W. Q. Zaman , L. M. Cao , J. Yang , ACS Appl. Mater. Interfaces 2017, 9, 41855. 29148711 10.1021/acsami.7b12775

[16] B. You , M. T. Tang , C. Tsai , F. Abild‐Pedersen , X. Zheng , H. Li , Adv. Mater. 2019, 31, 1807001. 10.1002/adma.201807001 30773741

[17] S. Sun , X. Zhou , B. Cong , W. Hong , G. Chen , ACS Catal. 2020, 10, 9086.

[18] H. Kim , N. A. Charipar , J. Figueroa , N. S. Bingham , A. Piqué , AIP Adv. 2019, 9, 015302.

[19] H. S. Oh , H. N. Nong , T. Reier , A. Bergmann , M. Gliech , J. Ferreira de Araujo , E. Willinger , R. Schlogl , D. Teschner , P. Strasser , J. Am. Chem. Soc. 2016, 138, 12552. 27549910 10.1021/jacs.6b07199

[20] A. Kumar , X. Liu , J. Lee , B. Debnath , A. R. Jadhav , X. Shao , V. Q. Bui , Y. Hwang , Y. Liu , M. G. Kim , H. Lee , Energy Environ. Sci. 2021, 14, 6494.

[21] N. B. Halck , V. Petrykin , P. Krtil , J. Rossmeisl , Phys. Chem. Chem. Phys. 2014, 16, 13682. 24671166 10.1039/c4cp00571f

[22] a) T. Shinagawa , A. T. Garcia‐Esparza , K. Takanabe , Sci. Rep. 2015, 5, 13801; 26348156 10.1038/srep13801 PMC4642571

[23] D. Böhm , M. Beetz , M. Schuster , K. Peters , A. G. Hufnagel , M. Döblinger , B. Böller , T. Bein , D. Fattakhova‐Rohlfing , Adv. Funct. Mater. 2020, 30, 1906670.

[24] a) K. A. Stoerzinger , O. Diaz‐Morales , M. Kolb , R. R. Rao , R. Frydendal , L. Qiao , X. R. Wang , N. B. Halck , J. Rossmeisl , H. A. Hansen , T. Vegge , I. E. L. Stephens , M. T. M. Koper , Y. Shao‐Horn , ACS Energy Lett. 2017, 2, 876;

[25] T. Liu , S. Yang , J. Guan , J. Niu , Z. Zhang , F. Wang , Small Methods 2022, 6, 2101156. 10.1002/smtd.202101156 35041267

[26] S. Hao , H. Sheng , M. Liu , J. Huang , G. Zheng , F. Zhang , X. Liu , Z. Su , J. Hu , Y. Qian , L. Zhou , Y. He , B. Song , L. Lei , X. Zhang , S. Jin , Nat. Nanotechnol. 2021, 16, 1371. 34697492 10.1038/s41565-021-00986-1

[27] M. J. Hÿch , L. Potez , Philos. Mag. A 1997, 76, 1119.

[28] M. Wang , Z. Feng , Curr. Opin. Electrochem. 2021, 30, 100803.

[29] L.‐J. Meng , M. P. dos Santos , Thin Solid Films 2000, 375, 29.

[30] D. Rochefort , P. Dabo , D. Guay , P. M. A. Sherwood , Electrochim. Acta 2003, 48, 4245.

[31] H. S. Oh , H. N. Nong , T. Reier , M. Gliech , P. Strasser , Chem. Sci. 2015, 6, 3321. 28706696 10.1039/c5sc00518c PMC5490338

[32] a) G. Kresse , J. Furthmüller , Comput. Mater. Sci. 1996, 6, 15;

[33] J. K. Nørskov , J. Rossmeisl , A. Logadottir , L. Lindqvist , J. R. Kitchin , T. Bligaard , H. Jonsson , J. Phys. Chem. B 2004, 108, 17886. 10.1021/jp047349j 39682080

