# Supplementary material for: Tensile‐Strained RuO2 Loaded on Antimony‐Tin Oxide by Fast Quenching for Proton‐Exchange Membrane Water Electrolyzer
Source: Adv Sci (Weinh). 2022 Jun 19;9(23):2201654. doi: 10.1002/advs.202201654 (PMC9376819; doi:10.1002/advs.202201654)
Supplement: Supplementary file 1 — Supporting Information [file ADVS-9-2201654-s001.pdf]

Supporting information

Tensile-Strained RuO_2 Loaded on Antimony-Tin Oxide by Fast Quenching for Proton-Exchange Membrane Water Electrolyzer

*Bing Huang, Hengyue Xu, Nannan Jiang, Minghao Wang, Jianren Huang, and Lunhui Guan**

B. Huang, N. Jiang, M. Wang, J. Huang, Prof. L. Guan
CAS Key Laboratory of Design and Assembly of Functional Nanostructures
Fujian Key Laboratory of Nanomaterials
Fujian Institute of Research on the Structure of Matter
Chinese Academy of Sciences
Fuzhou, 350000, China
*E-mail: guanlh@fjirsm.ac.cn

H. Xu
Institute of Biopharmaceutical and Health Engineering
Tsinghua Shenzhen International Graduate School
Tsinghua University
Shenzhen 518055, China

B. Huang, N. Jiang
University of Chinese Academy of Sciences
Beijing, 100049, China

Keywords: Electrochemical catalysis, RuO_2, Water electrolysis, Oxygen evolution reaction, Proton-exchange membrane water electrolyzer

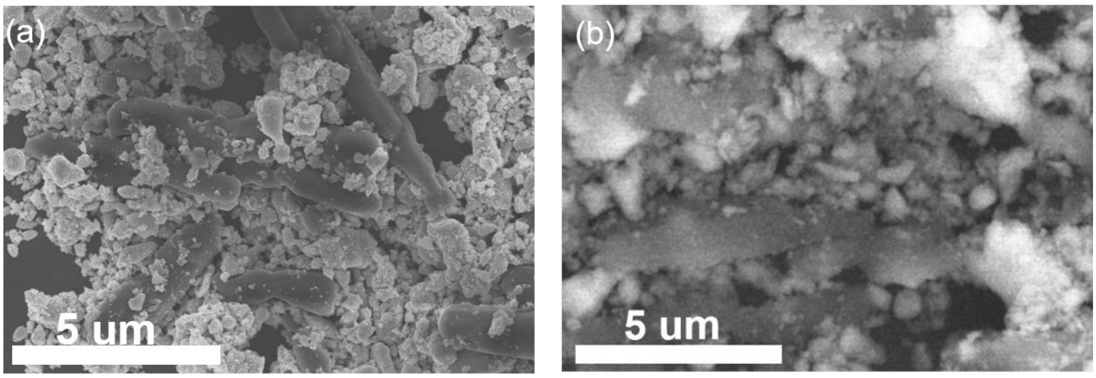

Figure S1. The SEM images of Co-HMT/ATO. a) The SEM image recorded at secondary electron imaging mode. b) The SEM image recorded at backscattered electron imaging mode. The lighter regions in Figure S1b correspond to heavier elements: Sn and Sb.

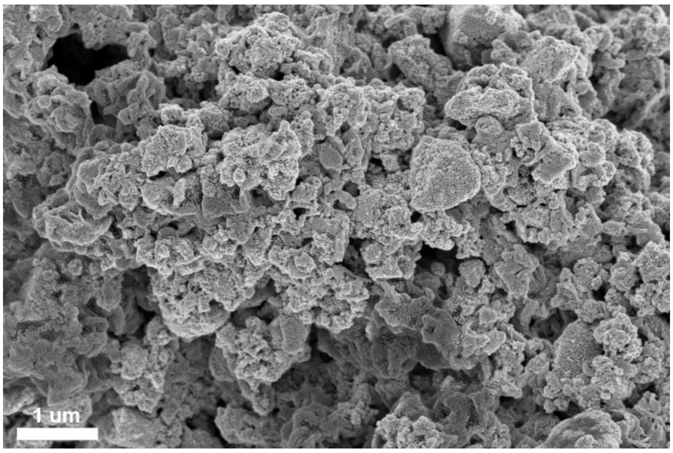

Figure S2. The SEM image of Ru-Co-HMT/ATO.

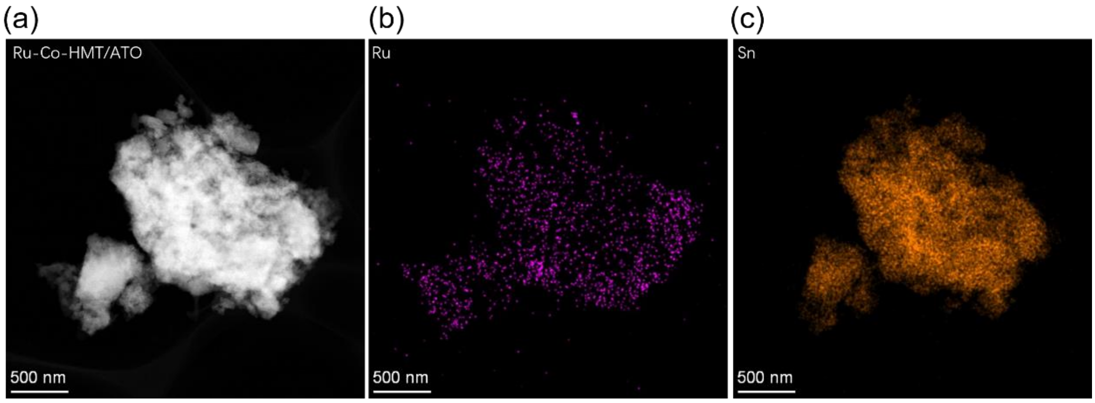

Figure S3. a) The high-angle annular dark-field (HAADF) image of Ru-Co-HMT/ATO. The lighter regions correspond to the ATO substrate due to its higher Z contrast. As can be seen, the ATO is surrounded by a darker layer, which is the amorphous Ru-Co-HMT.

b)-c) The elements mapping images of Ru-Co-HMT/ATO. As shown in it, the distribution of Ru is observed to be broader than Sn, proving the Ru has resided in the amorphous Ru-Co-HMT layer.

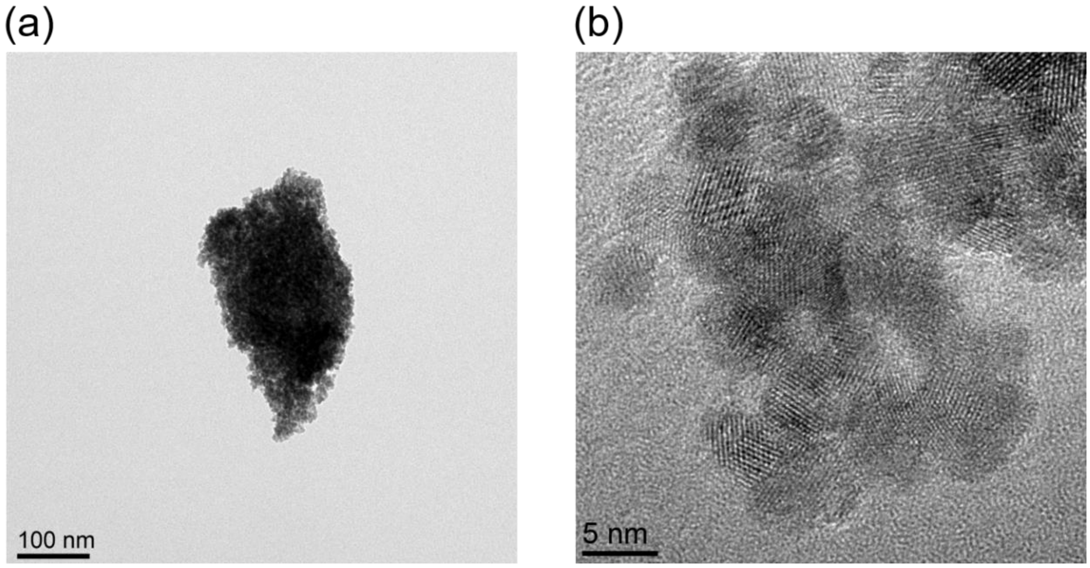

Figure S4. a) The TEM image of ATO. b) The high-resolution TEM image of ATO. As can be seen, the ATO consists of nanoparticles smaller than 10 nm.

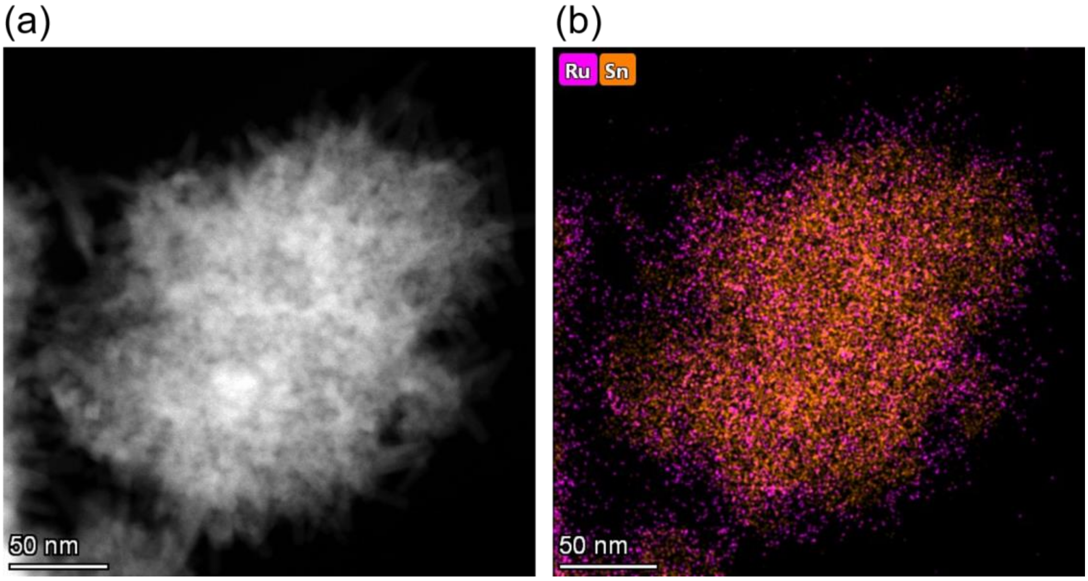

Figure S5. a) The high-angle annular dark-field (HAADF) image of *s*-RuO_2/ATO. b)-c) The elements mapping images of *s*-RuO_2/ATO.

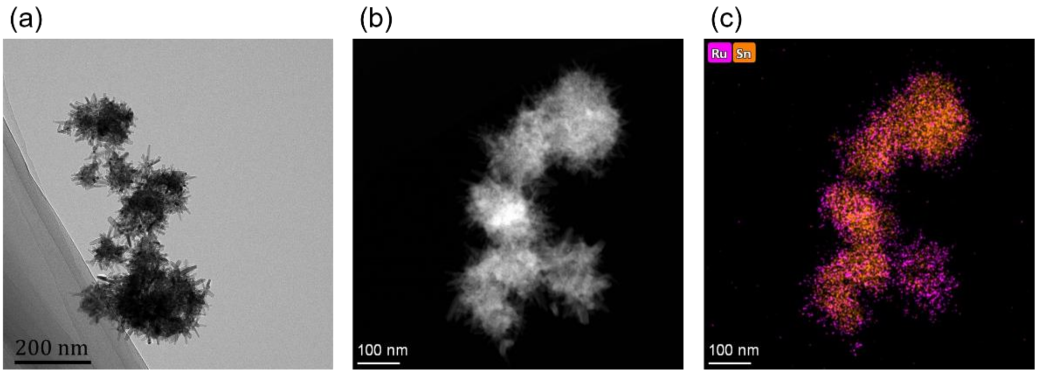

Figure S6. a) The TEM image of *n*-RuO_2/ATO. b) The HAADF image of *n*-RuO_2/ATO. c) The elements mapping images of Ru and Sn.

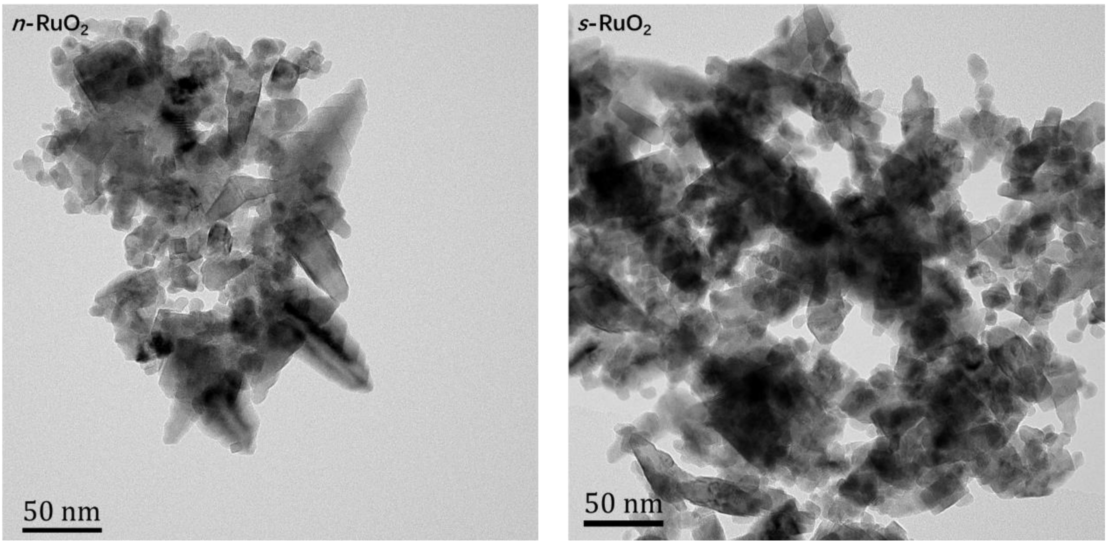

Figure S7. The TEM images of *n*-RuO_2 and *s*-RuO_2. As can be seen, part of the nanorods have sizes even broader than 30 nm.

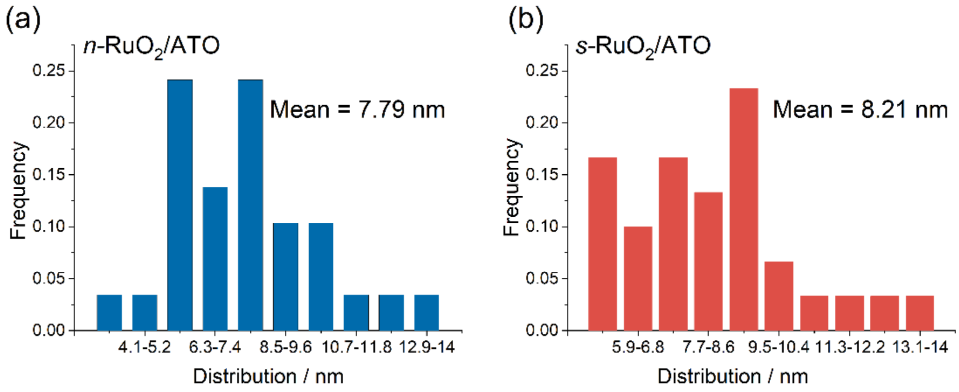

Figure S8. The nanorod widths of *n*-RuO_2/ATO and *s*-RuO_2/ATO. This data was obtained by counting 30 nanorods.

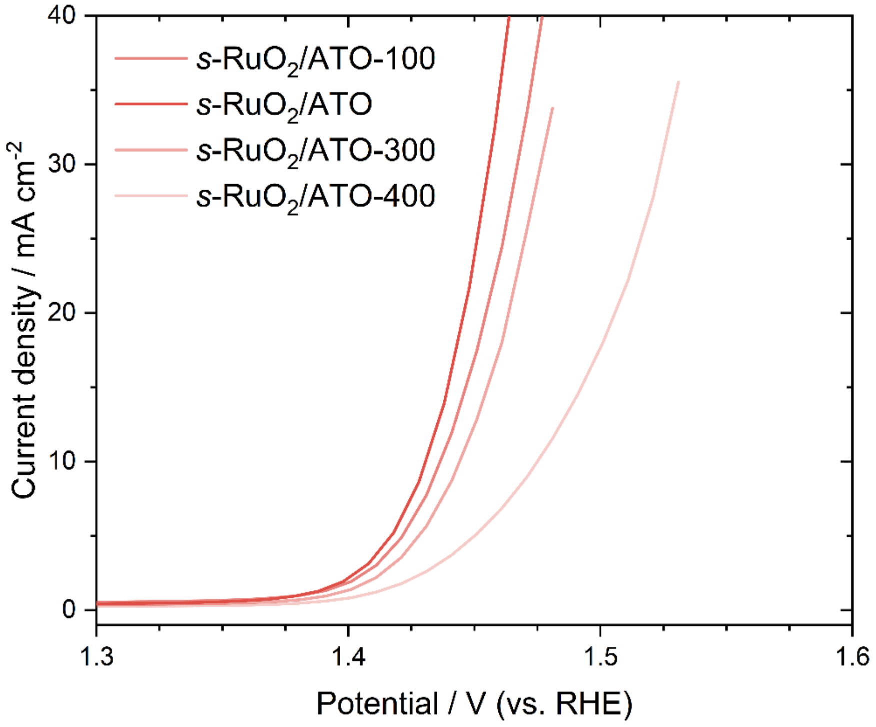

Figure S9. The LSV curves *s*-RuO_2/ATO with different ATO mass in the precursors from 100-400 mg ATO.

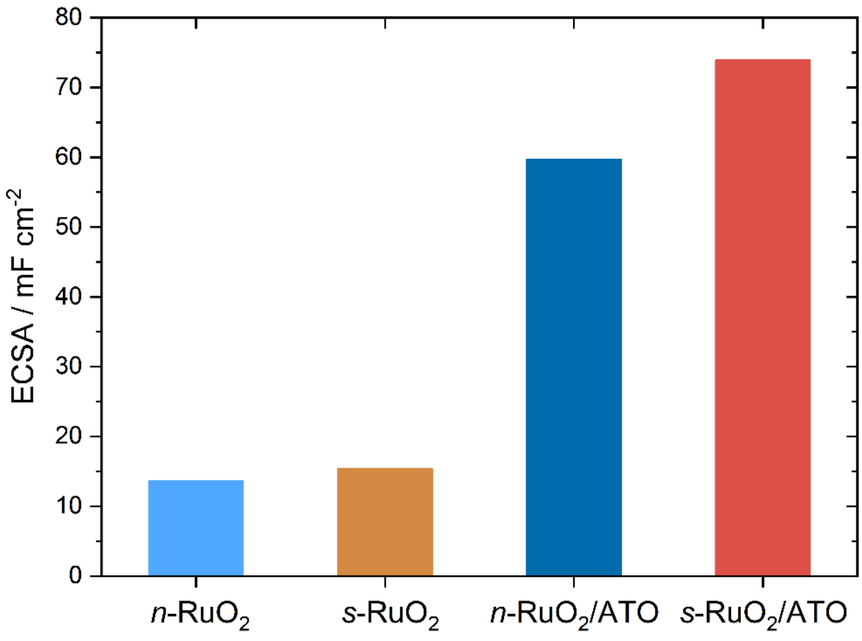

Figure S10. The ECSA of *n*-RuO_2, *s*-RuO_2, *n*-RuO_2-ATO, and *s*-RuO_2/ATO. The ECSA is calculated by: $\mathrm{ECSA} = \frac{\int \mathrm{vdi}}{\upsilon}$, where the v, i, and υ are voltage, current, and scan rate.

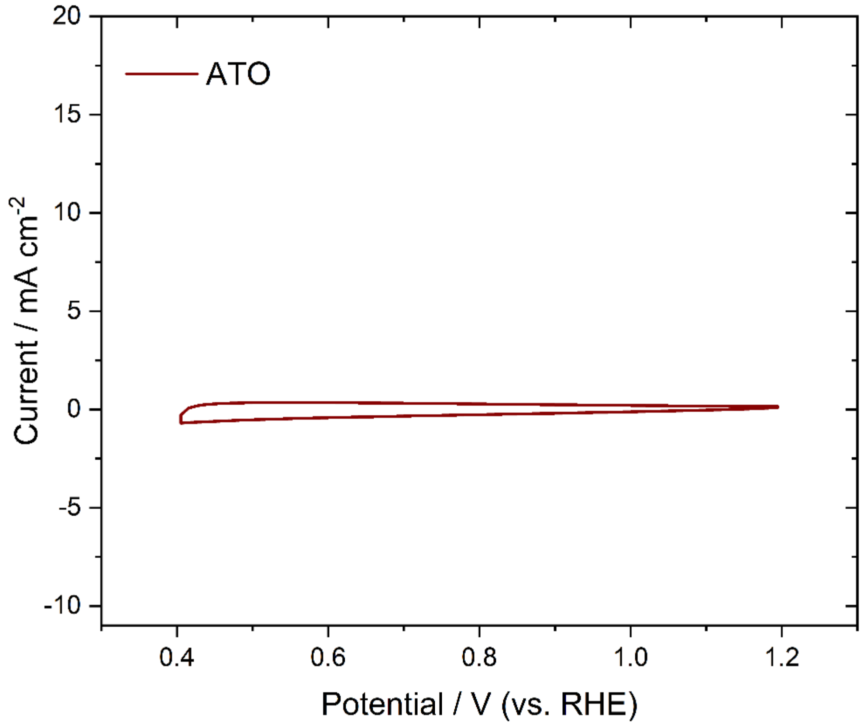

Figure S11. The CV curves of ATO. The load mass is 0.75 mg cm^{-2}, equal to the ATO fractions in *s*-RuO_2/ATO and *n*-RuO_2/ATO.

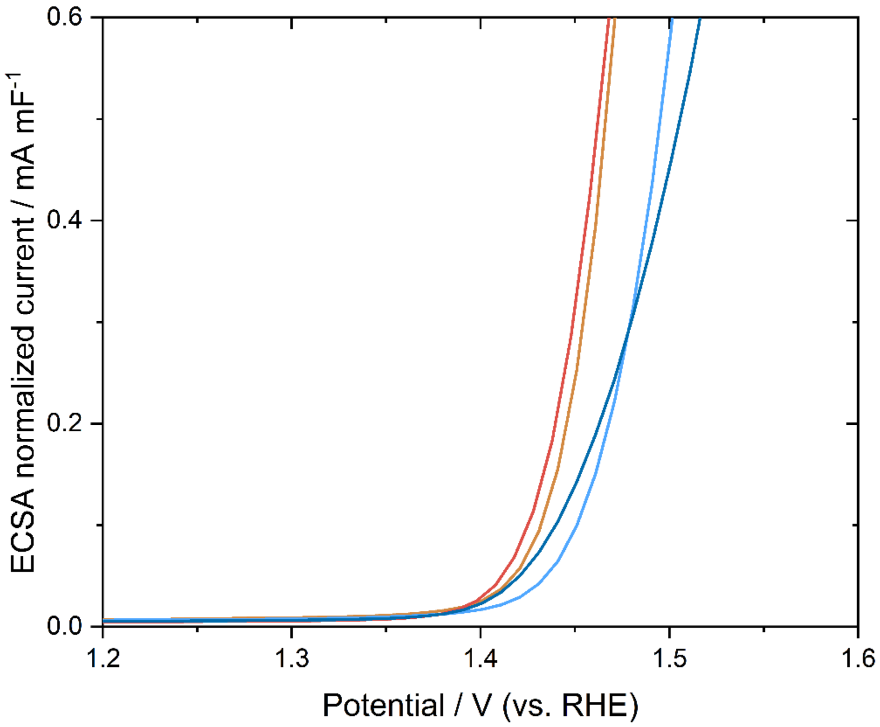

Figure S12. The ECSA Normalized LSV curves of *n*-RuO_2, *s*-RuO_2, *n*-RuO_2-ATO, and *s*-RuO_2/ATO.

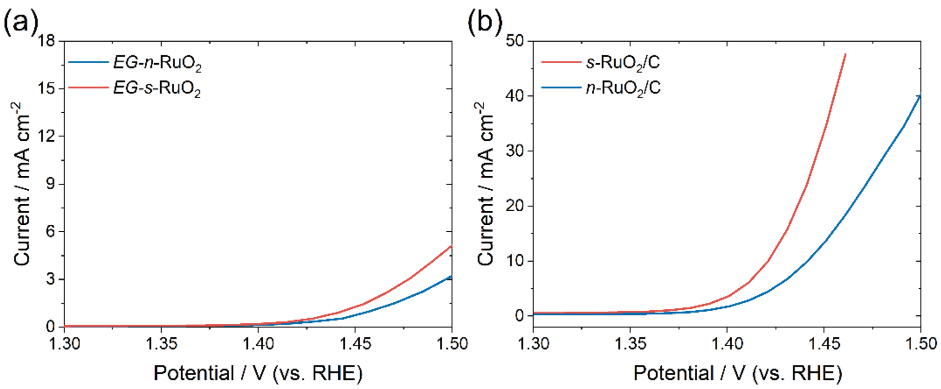

Figure S13. a) The LSV curves of *EG*-*n*-RuO_2 and *EG*-*s*-RuO_2. b) The LSV curves of *n*-RuO_2/C and *s*-RuO_2/C.

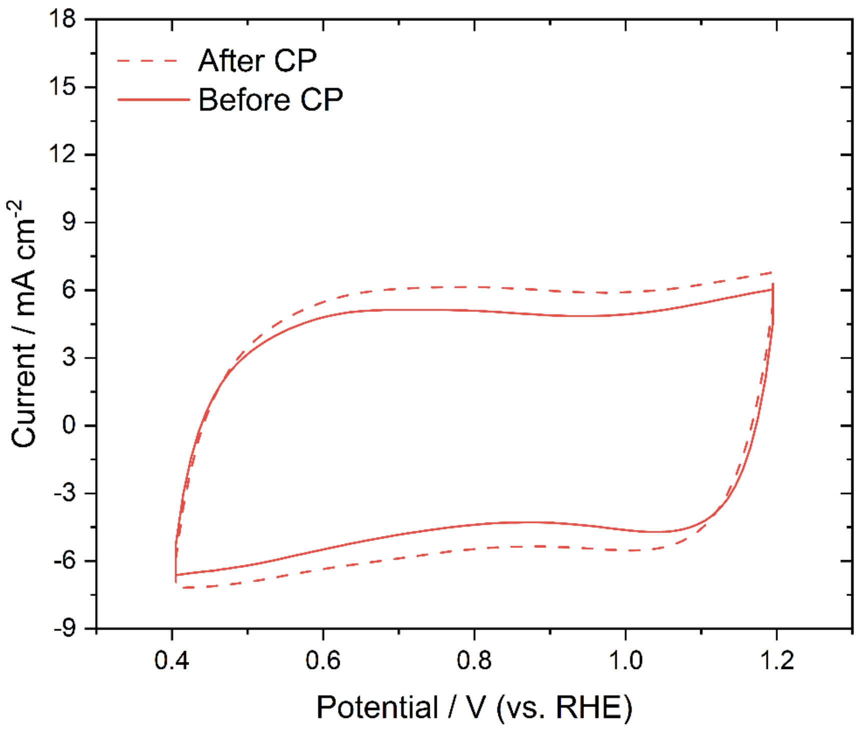

Figure S14. The CV curves of *s*-RuO_2/ATO before and after 12 h chronopotentiometry test.

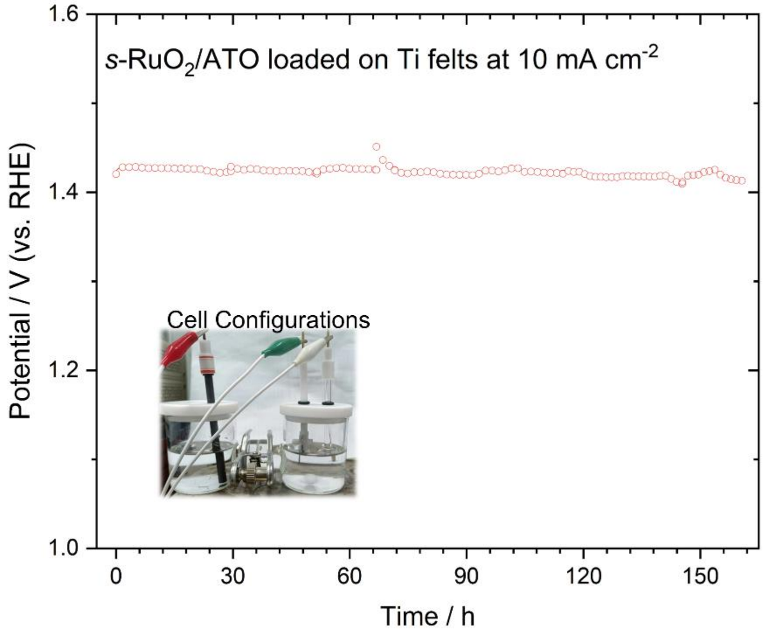

Figure S15. The long-term chronopotentiometry of s-RuO2/ATO tested on Ti felt. The electrolyte, ink preparation, load mass of *s*-RuO_2/ATO, and reference electrode were the same as the RDE test, except an H-cell was used. A Nafion membrane (Nafion 212) was also used to separate the anode and cathode cell apartments.

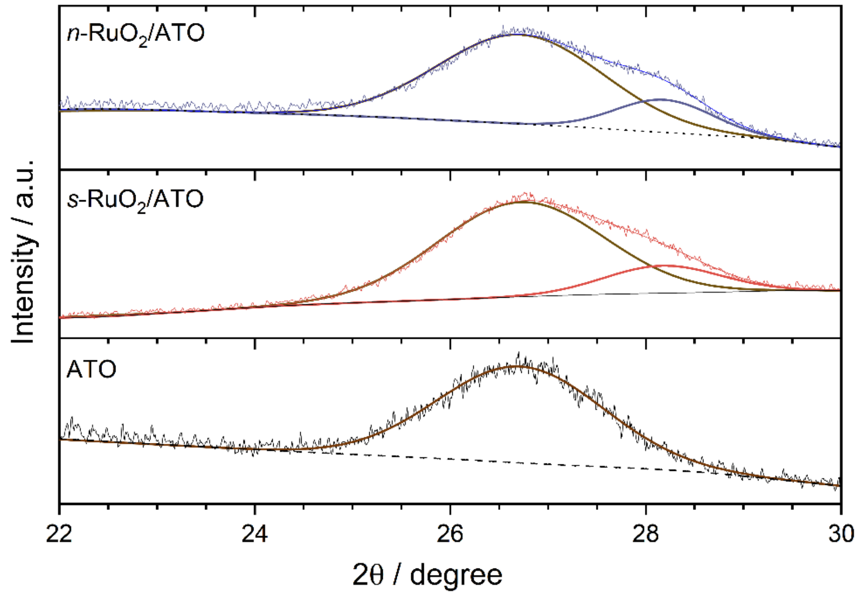

Figure S16. The Fitted XRD patterns (22-30 degree) of *n*-RuO_2-ATO, *s*-RuO_2/ATO, and ATO. All the ATO peaks were set to be fixed in positions and FWHMs.

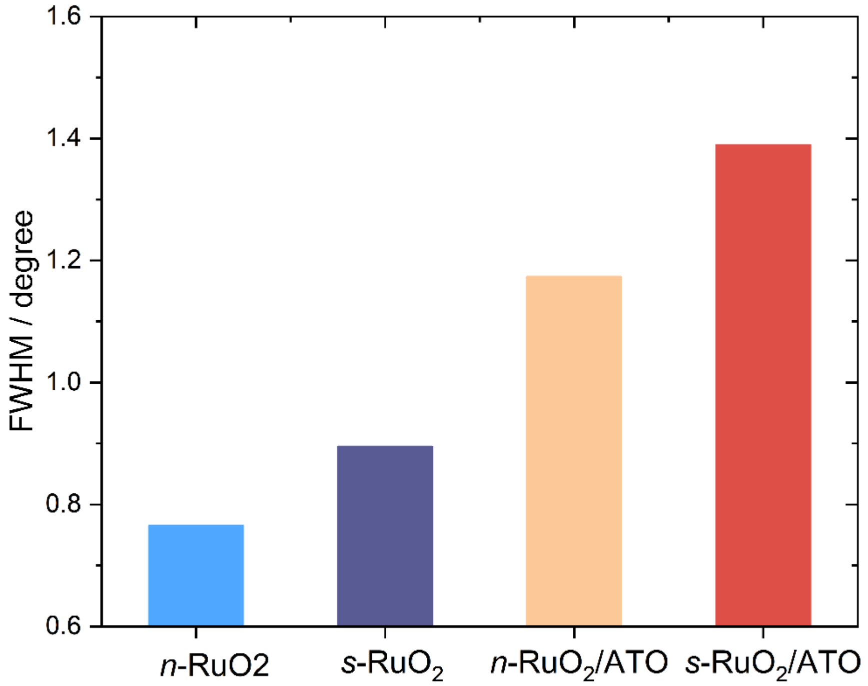

Figure S17. The FWHM of ruthenium oxide peaks (110) for *n*-RuO_2, *s*-RuO_2, *n*-RuO_2-ATO, and *s*-RuO_2/ATO. Here, we should note that existing tensile strains would also broaden the peaks.

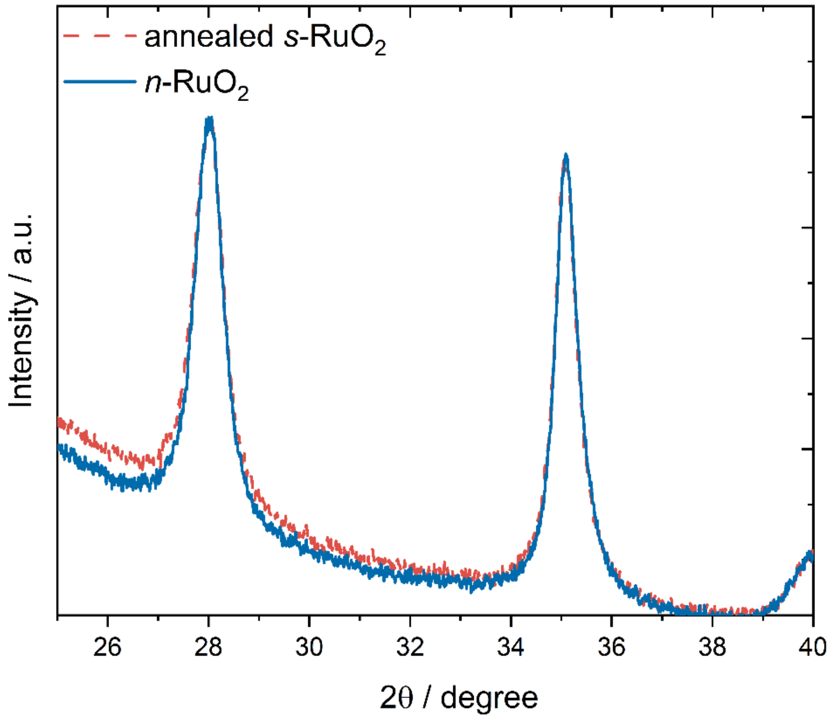

Figure S18. The XRD patterns of the annealed *n*-RuO_2 and the annealed *s*-RuO_2. Notably, the FWHMs and peak positions of the annealed *s*-RuO_2 return identical to *n*-RuO_2.

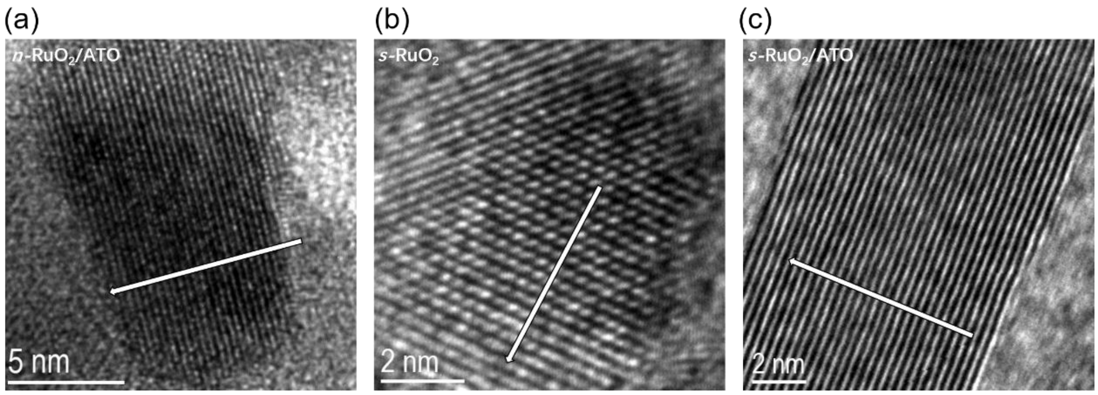

Figure S19. a) TEM images of *n*-RuO_2/ATO, *s*-RuO_2, and *s*-RuO_2/ATO. The inserted arrows are vertical to 110 planes.

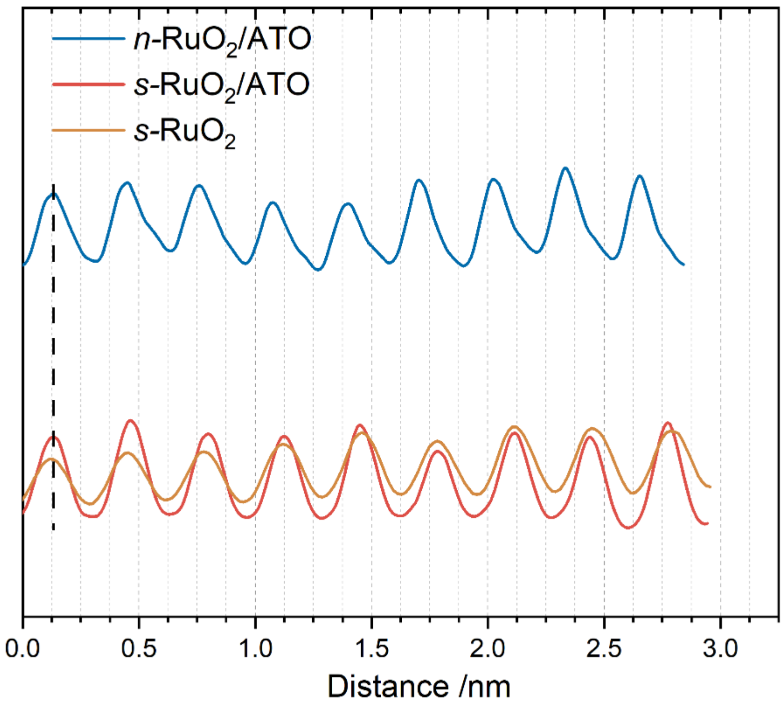

Figure S20. The intensity profiles alone the inserted arrow directions in figure S19. The average planear distances are 3.20, 3.23, and 3.23 Å for *n*-RuO_2/ATO, *s*-RuO_2/ATO, and *s*-RuO_2.

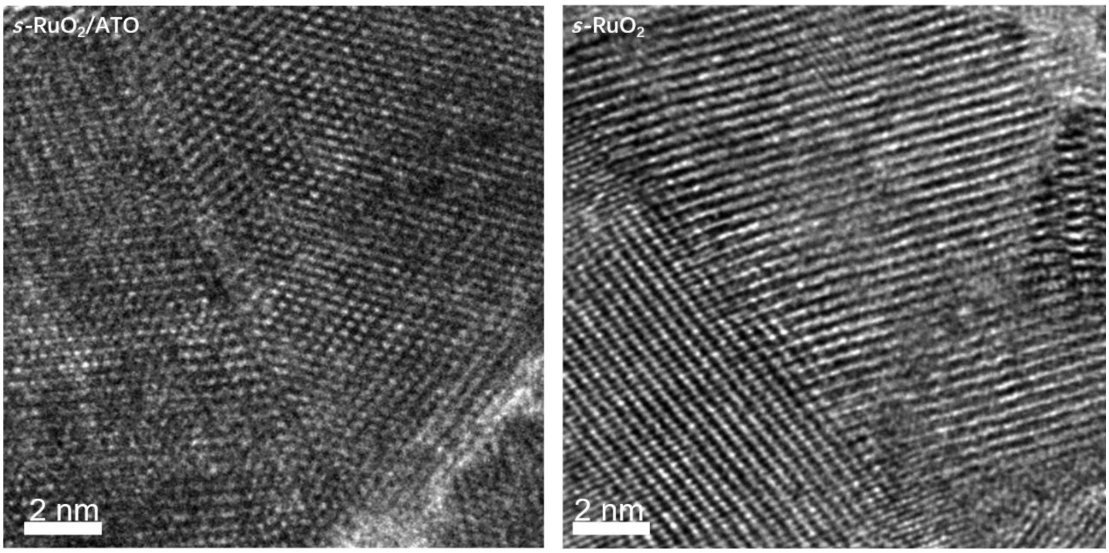

Figure S21. The HRTEM images of *s*-RuO_2/ATO and *s*-RuO_2. Grain boundaries can be observed in them.

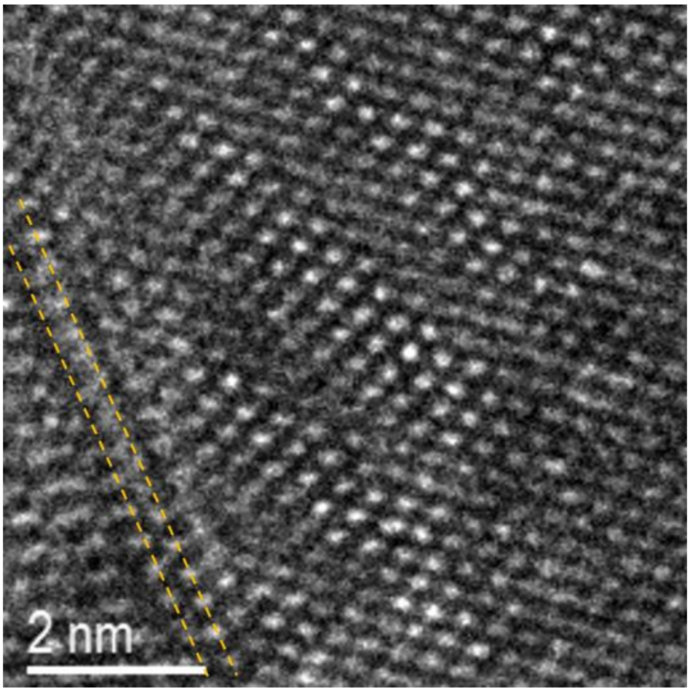

Figure S22. The high-resolution TEM image of *s*-RuO_2/ATO used for GPA.

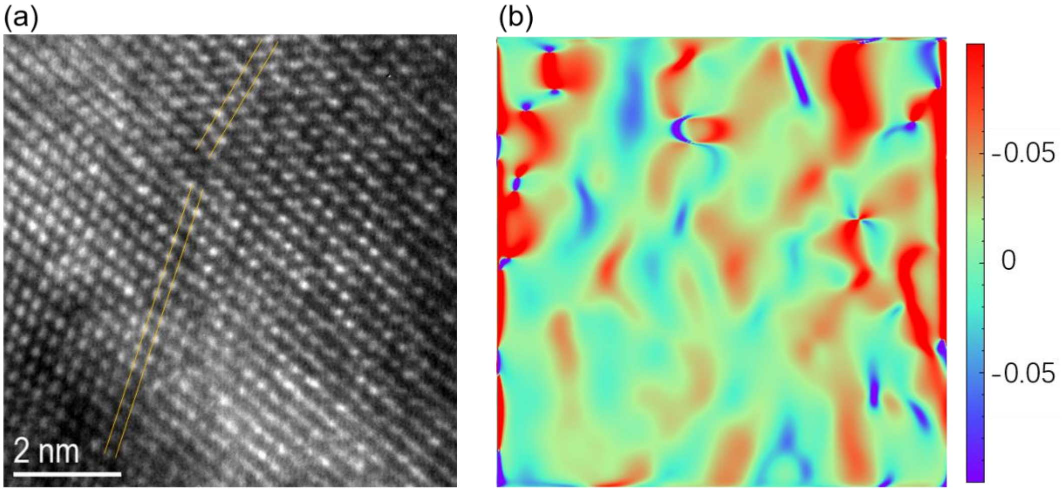

Figure S23. a) The high-resolution TEM image of *s*-RuO_2 used for GPA. b) the axial strain distribution obtained by GPA. The yellow lines in (a) indicate the stacking fault.

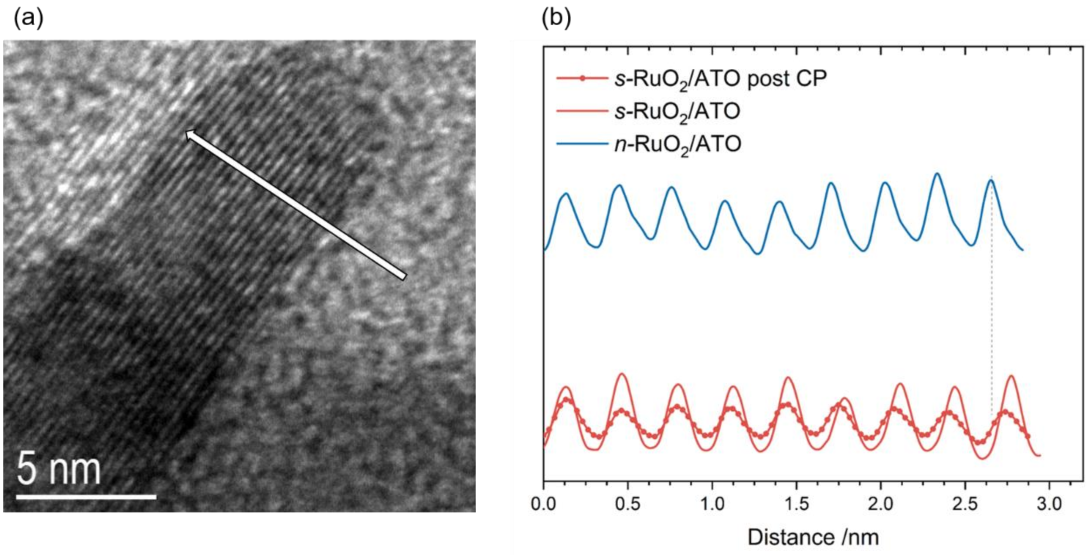

Figure S24. a) The high-resolution TEM image of *s*-RuO_2/ATO after chronopotentiometry. b) the intensity profile along the inserted arrow direction in S24a. For comparison, intensity profiles in **Figure S20** were also plotted here. The average interplanar distance of *s*-RuO_2/ATO post CP is 3.22 Å.

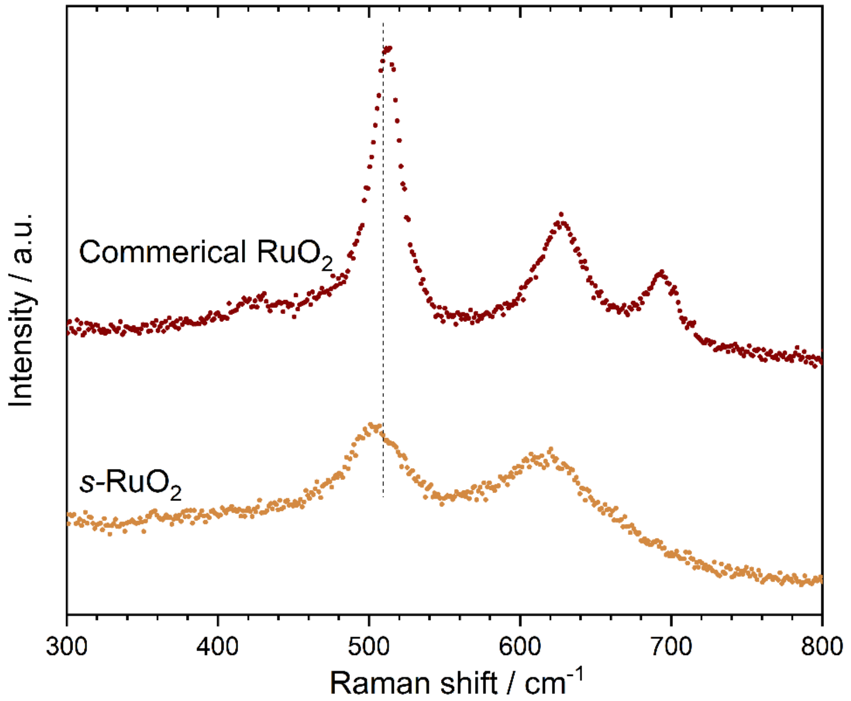

Figure S25. The Raman spectra of *s*-RuO_2 and Commercial RuO_2.

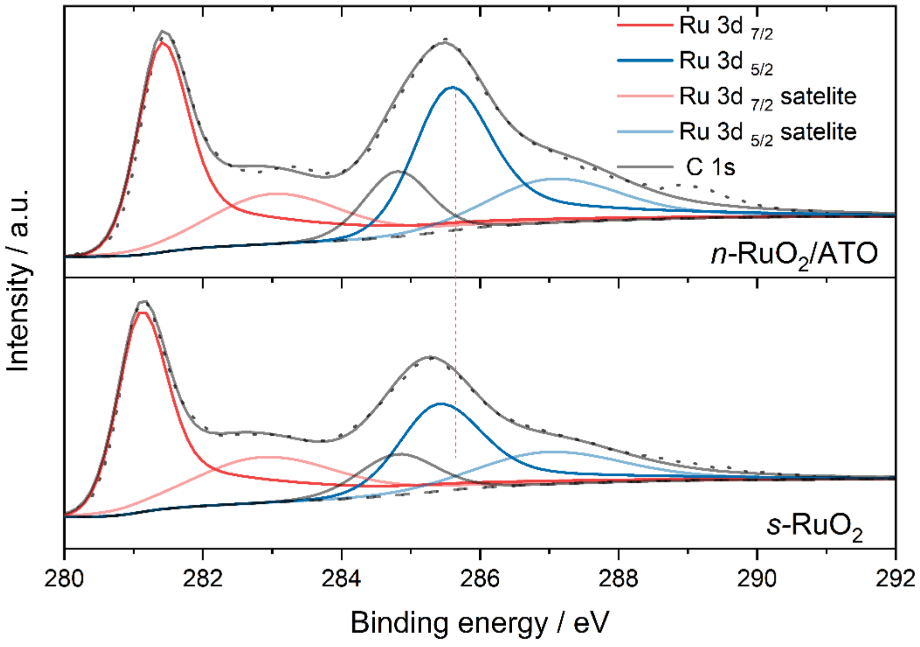

Figure S26. The XPS spectra of *n*-RuO_2/ATO and *s*-RuO_2.

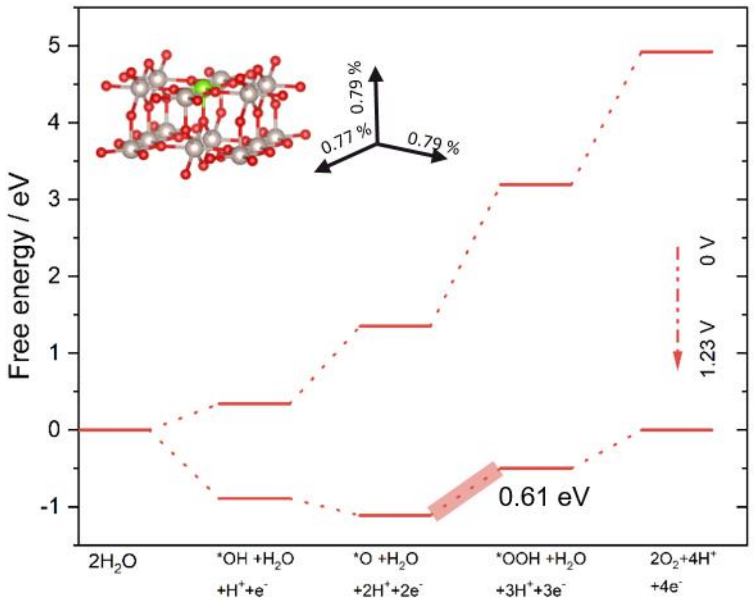

Figure S27. The free energy diagram of the strained RuO_2 with full coverage of bridge-O.

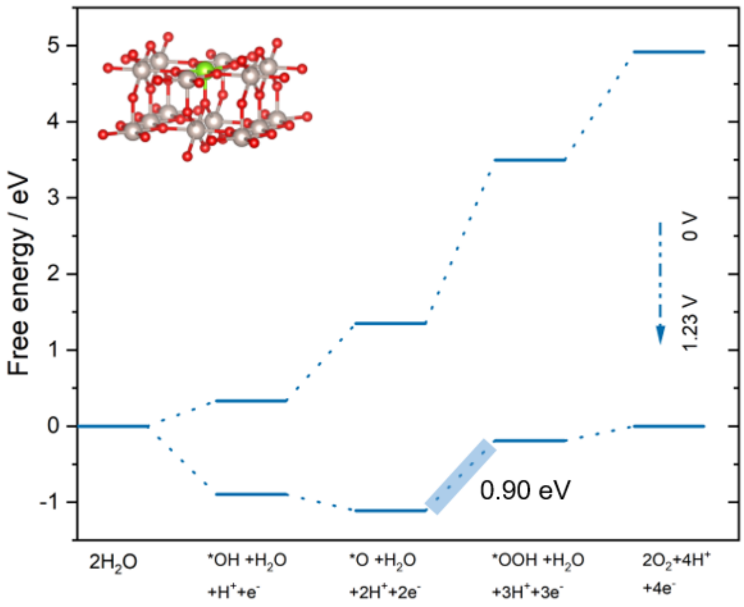

Figure S28. The free energy diagram of the RuO_2 with full coverage of bridge-O.

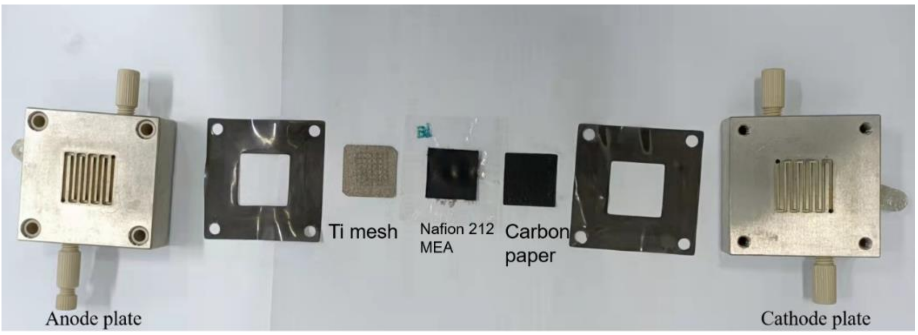

Figure S29. The real picture of our PEMWE.

Table S1. The elemental weight ratios from ICP-MS.

Samples	Ru (wt%)	Co (wt%)
Co-HMT	-/-	16.9%
Co-HMT/ATO	-/-	9.7%
n-RuO_2/ATO	17.2	2.8
s-RuO_2/ATO	19.4	2.4

n-RuO_2	63.1	7.2
s-RuO_2	64.0	6.4

Table 2. The previous reports related to Ru OER.

Samples	Electrolytes	η_{10} (mV)	References
Amorphous-RuO_2	0.1 M $HClO_4$	205	*Angew. Chem. Int. Ed.* **2021**, *60*, 18821-18829.
Co-doped RuO_2	1 M KOH	200	*Angew. Chem. Int. Ed.* **2022**, *61*, e202114951.
RuO_2/$(Co,Mn)_3O_4$	0.5 M H_2SO_4	270	*Appl. Catal. B* **2021**, *297.*
PtCo coated RuO_2/C	0.1 M $HClO_4$	212	*Energy Environ. Sci., 2022, 15, 1119–1130*
SrRuIr ternary oxide	0.5 M H_2SO_4	190	*J. Am. Chem. Soc.* **2021**, *143*, 6482-6490.
RuO_2 Nano sheets	0.5 M H_2SO_4	199	*Energy Environ. Sci.* **2020**, *13*, 5143-5151.
Co-doped RuO_2	0.5 M H_2SO_4	169	*iScience* **2020**, *23*, 100756.
Ru single atoms on γ-MnO_2	0.1 M $HClO_4$	161	*Nat. Catal.* **2021,** *4, 1012-1023.*
Pt-doped RuO_2	0.5 M H_2SO_4	228	*Sci. Adv.* **2022**, *8*, eabl9271.
s-RuO_2/ATO	0.1 M $HClO_4$	198	This work

Table S3. The Rietveld refinement results.

Parameters	*n*-RuO_2	*s*-RuO_2
R_{wp}	4.23%	2.66%
R_p	3.09%	2.09%
a (Å)	4.518	4.554

b (Å)	4.518	4.554
c (Å)	3.110	3.134
Interplane distances (1 1 0) (Å)	3.194	3.220

Table S4. The calculated free energies.

	$2H_2O$	$*OH+H_2O$ $+H^++e^-$	$*O+H_2O$ $+2H^++2e^-$	$*OOH$ $+3H^++3e^-$	$2O_2$ $+4H^++4e^-$
RuO_2	0 eV	-0.01 eV	0.92 eV	2.84 eV	4.92 eV
strained RuO_2	0 eV	0.01 eV	0.93 eV	2.88 eV	4.92 eV
RuO_2 with bridge O	0 eV	0.33 eV	1.35 eV	3.49 eV	4.92 eV
strained RuO_2 with bridge O	0 eV	0.34 eV	1.35 eV	3.20 eV	4.92 eV
